# Comparison of hypofractionation and standard fractionation for post-prostatectomy salvage radiotherapy in patients with persistent PSA: single institution experience

**DOI:** 10.1186/s13014-021-01808-3

**Published:** 2021-05-12

**Authors:** Jure Murgic, Blanka Jaksic, Marin Prpic, Davor Kust, Amit Bahl, Mirjana Budanec, Angela Prgomet Secan, Pierfrancesco Franco, Ivan Kruljac, Borislav Spajic, Nenad Babic, Bozo Kruslin, Mario Zovak, Eduardo Zubizarreta, Eduardo Rosenblatt, Ana Fröbe

**Affiliations:** 1 Department of Oncology and Nuclear Medicine, University Hospital Center Sestre Milosrdnice, Vinogradska 29, 10000 Zagreb, Croatia; 2 School of Dental Medicine, University of Zagreb, Gunduliceva 5, 10000 Zagreb, Croatia; 3 University Hospitals Bristol NHS Foundation Trust, Marlborough Street, Bristol, BS13NU UK; 4 Department of Translational Medicine, University of Eastern Piedmont, 28100 Novara, Italy; 5 Department of Radiation Oncology, ‘Maggiore della Carità’ University Hospital, 28100 Novara, Italy; 6 Department of Endocrinology, Diabetes and Metabolic Diseases “Mladen Sekso”, University Hospital Center Sestre Milosrdnice, University of Zagreb School of Medicine, Vinogradska 29, 10000 Zagreb, Croatia; 7 Department of Urology, University Hospital Center Sestre Milosrdnice, 10000 Zagreb, Croatia; 8 Department of Radiology, University Hospital Center Sestre Milosrdnice, Vinogradska 29, 10000 Zagreb, Croatia; 9 Ljudevit Jurak Department of Pathology and Cytology, Sestre Milosrdnice University Hospital Centre, Vinogradska 29, 10000 Zagreb, Croatia; 10 Department of Surgery, University Hospital Center Sestre Milosrdnice, Vinogradska 29, 10000 Zagreb, Croatia; 11 Division of Human Health, International Atomic Energy Agency (IAEA), Wagramer Str. 5, 1220 Vienna, Austria

**Keywords:** Radical prostatectomy, Prostate cancer, Salvage radiotherapy, Hypofractionation, Standard fractionation, Prostate-specific antigen persistence, Androgen deprivation therapy

## Abstract

**Background:**

Hypofractionated post-prostatectomy radiotherapy is emerging practice, however with no randomized evidence so far to support it’s use. Additionally, patients with persistent PSA after prostatectomy may have aggressive disease and respond less well on standard salvage treatment. Herein we report outcomes for conventionally fractionated (CFR) and hypofractionated radiotherapy (HFR) in patients with persistent postprostatectomy PSA who received salvage radiotherapy to prostate bed.

**Methods:**

Single institution retrospective chart review was performed after Institutional Review Board approval. Between May 2012 and December 2016, 147 patients received salvage postprostatectomy radiotherapy. PSA failure-free and metastasis-free survival were calculated using Kaplan–Meier method. Cox regression analysis was performed to test association of fractionation regimen and other clinical factors with treatment outcomes. Early and late toxicity was assessed using Common Terminology Criteria for Adverse Events (CTCAE) Version 4.0.

**Results:**

Sixty-nine patients who had persistent PSA (≥ 0.1 ng/mL) after prostatectomy were identified. Median follow-up was 67 months (95% CI 58–106 months, range, 8–106 months). Thirty-six patients (52.2%) received CFR, 66 Gy in 33 fractions, 2 Gy per fraction, and 33 patients (47.8%) received HFR, 52.5 Gy in 20 fractions, 2.63 Gy per fraction. Forty-seven (68%) patients received androgen deprivation therapy (ADT). 5-year PSA failure- and metastasis-free survival rate was 56.9% and 76.9%, respectively. Thirty patients (43%) experienced biochemical failure after salvage radiotherapy and 16 patients (23%) experienced metastatic relapse. Nine patients (13%) developed metastatic castration-resistant disease and died of advanced prostate cancer. Median PSA failure-free survival was 72 months (95% CI; 41–72 months), while median metastasis-free survival was not reached. Patients in HFR group were more likely to experience shorter PSA failure-free survival when compared to CFR group (HR 2.2; 95% CI 1.0–4.6, p = 0.04). On univariate analysis, factors significantly associated with PSA failure-free survival were radiotherapy schedule (CFR vs HFR, HR 2.2, 95% CI 1.0–4.6, p = 0.04), first postoperative PSA (HR 1.02, 95% CI 1.0–1.04, p = 0.03), and concomitant ADT (HR 3.3, 95% CI 1.2–8.6, p = 0.02). On multivariate analysis, factors significantly associated with PSA failure-free survival were radiotherapy schedule (HR 3.04, 95% CI 1.37–6.74, p = 0.006) and concomitant ADT (HR 4.41, 95% CI 1.6–12.12, p = 0.004). On univariate analysis, factors significantly associated with metastasis-free survival were the first postoperative PSA (HR 1.07, 95% CI 1.03–1.12, p = 0.002), seminal vesicle involvement (HR 3.48, 95% CI 1.26–9.6,p = 0.02), extracapsular extension (HR 7.02, 95% CI 1.96–25.07, p = 0.003), and surgical margin status (HR 2.86, 95% CI 1.03–7.97, p = 0.04). The first postoperative PSA (HR 1.04, 95% CI 1.00–1.08, p = 0.02) and extracapsular extension (HR 4.24, 95% CI 1.08–16.55, p = 0.04) remained significantly associated with metastasis-free survival on multivariate analysis.

Three patients in CFR arm (8%) experienced late genitourinary grade 3 toxicity.

**Conclusions:**

In our experience, commonly used hypofractionated radiotherapy regimen was associated with lower biochemical control compared to standard fractionation in patients with persistent PSA receiving salvage radiotherapy. Reason for this might be lower biological dose in HFR compared to CFR group. However, this observation is limited due to baseline imbalances in ADT use, ADT duration and Grade Group distribution between two radiotherapy cohorts. In patients with persistent PSA post-prostatectomy, the first postoperative PSA is an independent risk factor for treatment failure. Additional studies are needed to corroborate our observations.

## Background

Radiotherapy is a well-established treatment modality for recurrent prostate cancer following radical prostatectomy [1]. As surgery is increasingly being used as primary treatment for high-risk patients, the role of subsequent radiotherapy in optimizing patients’ outcomes becomes critical [2]. In patients with unfavorable pathological features found at prostatectomy (such as extracapsular disease, infiltration of seminal vesicles, Gleason score ≥ 8 or positive surgical margins), the incidence of biochemical failure can be as high as 60% at ten years post treatment [3–5]. Once the patient experience biochemical failure, defined as confirmed rise in PSA ≥ 0.2 ng/mL, the only available potentially curative treatment option is salvage radiotherapy [6–8].

Ideally, PSA should fall below 0.1 ng/mL 4–6 weeks after radical prostatectomy. However, substantial proportion of men fail to obtain undetectable PSA after radical prostatectomy. This scenario, termed as PSA persistence, is not uncommon event, particularly in high-risk patients [9].

Opposite to common assumption of incurability of such patients, in a recent much debated study the actuarial ten-year prostate cancer-specific survival was as high as 88% in cohort of patients who had PSA between 0.1 and 2.0 ng/mL two months post prostatectomy [10]. This finding highlighted the heterogeneity of such patient population and found that some patients have excellent outcomes while radiotherapy lead to clinical benefit only in patients who are at higher risk (≥ 30%) of prostate cancer-specific mortality based on developed multivariate model [11].

The standard way of delivering salvage radiotherapy is in 2 Gy fractions using conventionally fractionated radiotherapy (CFR), to total dose of ≥ 66 Gy [12]. However, success of recent hypofractionation (HFR) studies in definitive radiotherapy for intact prostate cancer provided rationale for use of hypofractionation in postoperative setting where such data are lacking [13–17].

Namely, prostate cancer exhibits unique radiobiological feature of high sensitivity to radiotherapy fraction size originating from its low alpha/beta, theoretically making HFR iso-effective or even superior to CFR, while sparing organs-at-risk which are deemed to have higher apha/beta ratio than prostate cancer [18, 19]. Recently, Lewis et al. reported early outcomes of their hypofractionation experience (fraction size 2.5 Gy to total dose 57.5–65 Gy) in patients receiving postprostatectomy salvage radiotherapy [20]. They found unusually high 4-year biochemical control of 75%, probably reflective of relatively short follow-up. Hypofractionation regimen (i.e. 52.5 Gy in 20 fractions) is common postoperative radiotherapy scheme. One third of patients randomized on RADICALS trial received such HFR [21]. Moreover, group from Christie Hospital recently published their experience with salvage HFR in 112 patients with 10 years of follow-up [22]. Authors externally validated Tendulkar nomogram [23], however no data on patients with persistent PSA were presented.

Global COVID-19 pandemic has set unprecedented challenges to radiation oncology never experienced so far in our field. ESTRO took position to address some of the growing issues [24]. One of the key areas affected is approach to fractionation as conventional fractionation is associated with increasing pressure on already present resource and labor force constraints. It is estimated the impact will be long lasting and will give rise to increasing use of hypofractionation even in postprostatectomy setting [25, 26].

The aim of our study was (1) to assess clinical outcomes of patients presenting with persistent and rising PSA after radical prostatectomy who received post-prostatectomy salvage radiotherapy and (2) to retrospectively compare two prostate bed salvage radiotherapy schedules: hypofractionated regimen (HFR) of 52.5 Gy in 20 fractions and conventionally fractionated regimen (CFR) of 66 Gy in 33 fractions, in regard to PSA failure-free and metastasis-free survival.

## Methods

### Patients characteristics

**Fig. 1:**
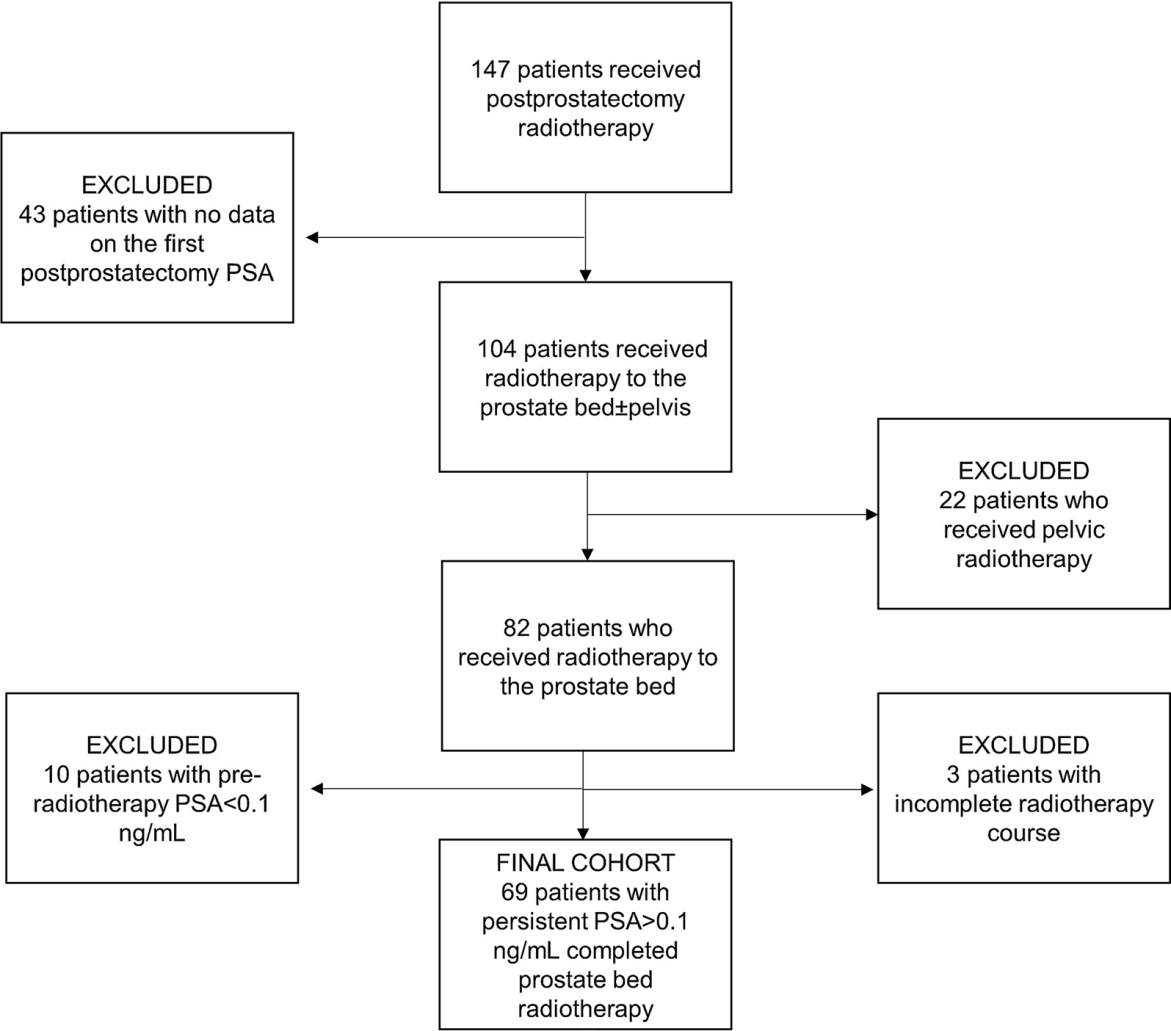
Consort diagram illustrating patient’s selection process

An audit of our prospectively updated database of patients treated with post-prostatectomy radiotherapy was performed. From 147 consecutive patients who received postprostatectomy radiotherapy in our institution Between May 2012 and December 2016, 81 patients had their first PSA measured 6–8 weeks after prostatectomy ≥ 0.1 ng/mL (among initial cohort of 147 patients, in 43 patients the information on first postoperative PSA was lacking). Twelve patients had pathologically involved pelvic lymph nodes and received additional pelvic radiotherapy in addition to prostate bed and were also excluded from this analysis. From remaining 69 patients, in 58 patients (84%) pelvic nodal dissection was not performed (hence pNx stage) and in 11 patients (16%) was performed. Among 11 patients who underwent pelvic nodal dissection, 6 patients were pN0, and 5 patients were pN1. The final cohort for this analysis consisted of 69 patients who received prostate bed salvage radiotherapy in a single tertiary center, regardless of pN status, had persistent PSA post prostatectomy, defined as ≥ 0.1 ng/mL measured 6–8 weeks after prostatectomy and had available information on follow-up in hospital records (see Consort diagram how patients were selected presented in Fig. 1).

Staging investigations included multiparametric MRI of the pelvis and/or choline PET/CT to assess local disease in the pelvis or detect distant metastatic spread. Androgen deprivation therapy (ADT) was prevalently used at the discretion of treating oncologist. Patients with at least two high-risk features present (i.e. positive margins and/or pT3b stage and/or PSA > 1 ng/mL) were offered ADT. The choice of the form of ADT (i.e. bicalutamide 150 mg vs LHRH agonist) and duration of ADT was decision of treating oncologist. In patients with several high risk features present (i.e. pT3b and/or Gleason score 8–10, and/or positive surgical margins and/or PSA > 1 ng/mL and/or presence of gross local recurrence revealed on imaging) the inclination was to prolong ADT duration to total of 2 years.

### Radiotherapy details

All patients had computerized tomography (CT) simulation done in supine position with full bladder and empty rectum. Simulation CT was done without intravenous contrast and slice thickness was 3 mm. Either leg and knee supporters (Combifix) or abdominal thermoplastic mask (Orifit) were used for patient immobilization. Clinical target volume (CTV) consisted of prostate bed plus extension to cover remnants of seminal vesicles according to EORTC postoperative radiotherapy for prostate cancer guidelines [27] with planning target volume (PTV) margin of 1 cm around CTV. Radiotherapy treatment planning was done in Xio software (Elekta) and employed 3-dimensional conformal technique using up to 8 noncoplanar high energy photon beams. Bladder and rectum were considered as organs-at-risk and were contoured as solid structures. Dose-volume histograms were generated for each plan and target dose objectives and constraints were used from updated Quantec report [28]. Radiotherapy was delivered on Elekta Synergy linear accelerator.

### Patient outcomes

Patients were followed every 3 months during first year after treatment, every 6 months for next 4 years and then annually after 5 years. Toxicity was retrospectively assessed through patient’s chart review by two investigators (JM, AF) using Common Terminology Criteria for Adverse Events (CTCAE) Version 4.0. Prostate-specific antigen (PSA) failure-free survival was measured from the date of the start of radiotherapy to the date of biochemical relapse, defined as a rise in PSA of 0.2 ng/mL above the postradiotherapy nadir followed by second higher value or any PSA value of more than 0.5 ng/mL above the nadir or initiation of salvage androgen deprivation therapy [29]. Metastasis-free survival was calculated from the radiotherapy start date to the date of imaging-confirmed metastatic relapse using any of the available imaging modalities (bone scan, computerized tomography, or choline PET/CT).

### Statistical analysis

Patient characteristics between the two cohorts were compared using one-way analysis of variance and chi-square methods for continuous and categorical variables, respectively. The Kaplan–Meier log-rank method was used to calculate PSA failure-free and metastasis-free survival for both radiotherapy fractionation schedules. Cox regression model (unadjusted and adjusted) was used to assess association of clinical and pathologic variables with the outcomes. Statistical analysis was performed using Medcalc version 19.1. statistical software and p-value of less than 0.05 was considered significant.

### Ethics

The approval of Institutional Ethics Committee was obtained for this study (Code EP-5992/17–9). This retrospective study was performed according to the principles of the Declaration of Helsinki (2008).

## Results

### Comparison of two radiotherapy fractionation regimens

Total of 69 patients with persistently elevated PSA post radical prostatectomy received prostate bed radiotherapy. Thirty-six patients (52%) received CFR of 66 Gy in 33 fractions (CFR) and 33 patients (48%) received HFR of 52.5 Gy in 20 fractions (HFR). The median follow-up for whole cohort was 67 months (95% CI 58–106 months). Table 1 shows characteristics of patients who received either CFR or HFR. There were few significant differences between CFR and HFR group in regard to baseline prognostic factors. Patients in CFR group were more likely to receive additional concomitant and adjuvant androgen deprivation therapy (ADT), either in the form of oral antiandrogen bicalutamide 150 mg or luteinizing hormone-releasing hormone (LHRH) agonist. More precisely, 81% of patients in CFR group compared to 55% in HFR group received additional ADT, respectively (p = 0.02). The reason behind this decision is the higher prevalence of patients with multiple risk factors in CFR group. Among patients in CFR group who received ADT, dominant form of hormonal therapy was bicalutamide 150 mg (55%), while in HFR group more prevalent were LHRH agonists (61%). Median duration of ADT in all cohort was 24 months, however many patients in CFR group had longer course of ADT compared to patients in HFR group despite the same median duration of 24 months (p = 0.04). Finally, two groups differed in Grade Group (GG) prostatectomy pathology distribution (p = 0.02). In CFR group there was more patients with low grade disease, while in HFR there was more patients with high grade pathology.

**Table 1:** Patient characteristics of persistent PSA patient final cohort stratified by the two radiotherapy fractionation schedules (N = 69)

	Persistent PSA salvage radiotherapy cohort (N = 69)	CFR group 66 Gy in 33 fractions (N = 36)	HFR group 52.5 Gy in 20 fractions (N = 33)	*p*-value
Age, yr, median (IQR)	63 (59–68)	63 (55–68)	64 (59–68)	0.90
Follow-up post RT, Months, median (IQR)	59 (49–74)	59 (50–73)	64 (46–76)	0.98
First post- prostatectomy PSA, ng/mL Median (IQR)	0.33 (0.16–1.57)	0.3 (0.14–1.5)	0.36 (0.2–2.4)	0.16
PreRT PSA, ng/mL, median (IQR)	0.56 (0.30–1.83)	0.55 (0.29–1.71)	0.60 (0.33–2.39)	0.25
PreRT PSA, ng/mL, range	0.1–30.0	0.12–12.5	0.10–30.0	
ADT during RT, N (%)	47 (68)	29 (81)	18 (55)	0.02
Bicalutamide 150 mg, N (%)	23 (49)	16 (55)	7 (39)	
LHRH agonist, N (%)	24 (51)	13 (45)	11 (61)	
Duration of ADT, Months, median (IQR)	24 (6–27)	24 (6–30)	24 (21–27)	0.04
Final pathology Grade Group (GG), N (%)				0.021
GG 1	3 (4)	3 (8)	/	
GG 2	27 (39)	11 (30)	166 (48)	
GG 3	21 (30)	13 (36)	8 (24)	
GG 4	8 (11)	6 (16)	2 (6)	
GG 5	10 (16)	3 (10)	7 (22)	
pT stage, N (%)				0.08
T1–T2a	7 (10)	6 (16)	1 (3)	
T2b–T2c	22 (32)	13 (36)	9 (27)	
T3–T4	40 (58)	17 (19)	23 (70)	
SVI, N (%)	33 (48)	12 (33)	11 (33)	1.0
Positive SM, N (%)	28 (41)	14 (39)	14 (42)	0.77
ECE, N (%)	32 (46)	13 (36)	19 (58)	0.08

*CFR* conventionally fractionated radiotherapy, *HFR* hypofractionated radiotherapy, *IQR* interquartile range, *RT* radiotherapy, *PSA* prostate-specific antigen, *ADT* androgen deprivation therapy, *LHRH* Luteinizing hormone-releasing hormone, *SVI* seminal vesicle invasion, *SM* surgical margins, *ECE* extracapsular extension

### Oncologic efficacy analysis

In all cohort (N = 69), after median follow-up of 67 months (95% CI 58–106 months, range, 8–106 months), 30 biochemical failures (43%) and 16 metastatic relapses (23%) were observed. Median PSA progression-free survival was 72 months (95% CI; 41–72 months). Five-year PSA failure-free survival was 56.9%.

**Fig. 2:**
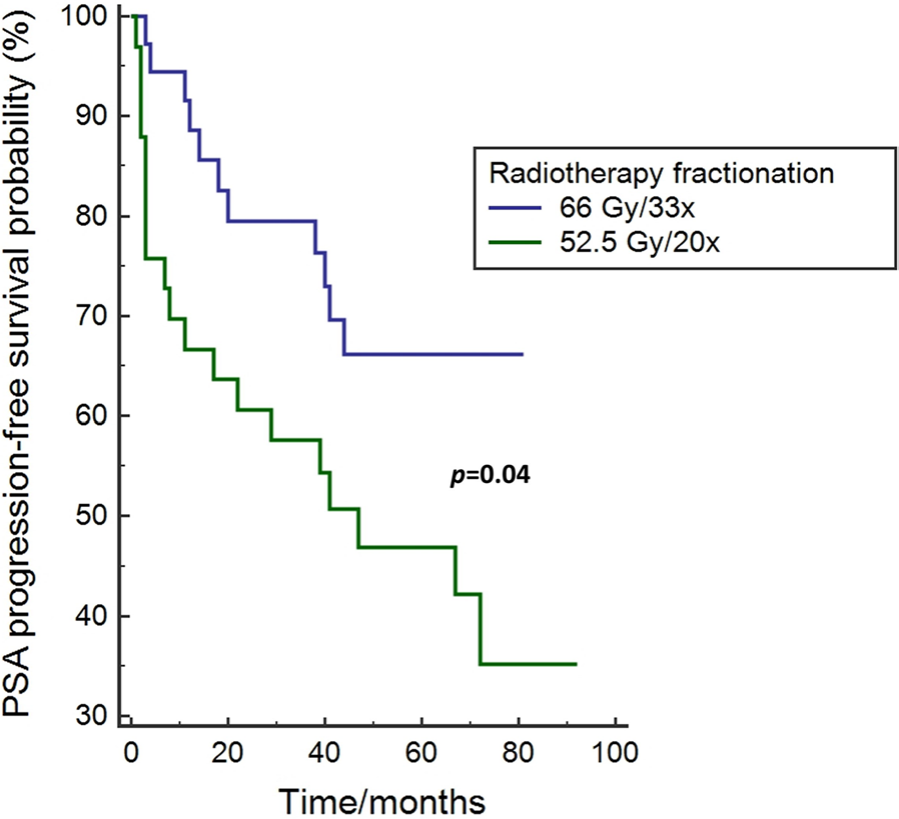
Kaplan–Meier curves for PSA failure-free survival for patients treated with salvage radiotherapy using two different fractionation regimens. Please note significant stratification of the curves (Log-rank *p* = 0.04)

When analyzed by fractionation schedule, median PSA relapse-free survival for CFR and HFR group were not reached and 47 months (95% CI 11–72 months), respectively (Log-rank *p* = 0.04) (Fig. 2).

**Fig. 3:**
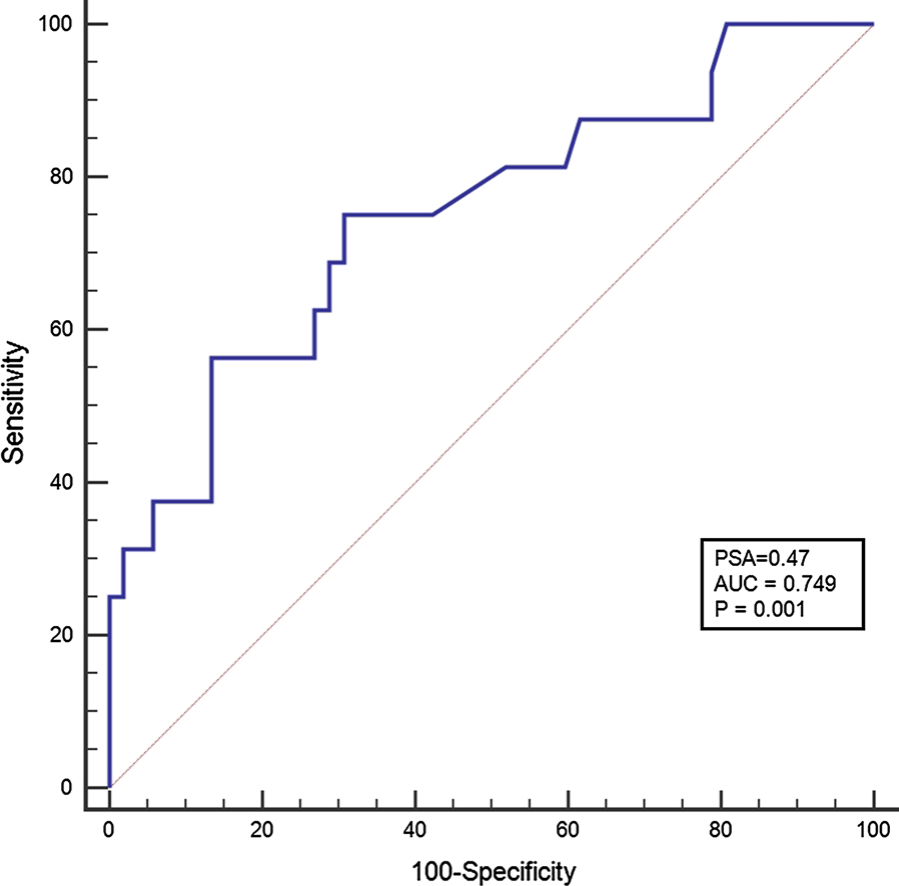
Receiver operating characteristic (ROC) curve analysis identified PSA = 0.47 ng/mL as optimal cut-off which discriminates metastatic relapse after salvage radiotherapy. Area under curve (AUC) 0.75 with sensitivity of 75%, specificity of 69%

In univariate analysis for biochemical failure using Cox hazard regression model, we tested association of the clinical and pathological variables with PSA progression-free survival (Table 2). We found statistically significant association of the first postprostatectomy PSA (entered as continuous variable), radiotherapy schedule, use of concomitant ADT, pT stage, and Grade Group with PSA progression-free survival. More precisely, patients with rising level of first postprostatectomy PSA, or patients treated with HFR, or patients who got ADT, or patients with higher pT stage or Grade Group were more likely to develop biochemical failure, i.e. to experience failure of salvage radiotherapy. The fact that we found the use of ADT to be associated with increased risk of PSA failure might be somehow surprising. Decision to add ADT to salvage radiotherapy was arbitrary and at discretion of treating oncologist, however, ADT was more frequently prescribed to patients with more adverse prognostic factors (i.e. combination of high PSA, positive surgical margins, extracapsular extension, more advanced T-stage, etc.).

**Table 2:** Univariate analysis for PSA failure-free survival after salvage radiotherapy

Variable	Hazard ratio (HR)	95% CI	*p*-value
Age, yrs Cont	1.01	0.95–1.08	0.99
First post- prostatectomy PSA Cont	1.02	1.0–1.04	0.03
PreRT PSA Cont	1.05	0.997–1.09	0.06
Fractionation schedule			
66 Gy/33x	1.0		
52.5 Gy/20x	2.2	1.0–4.6	0.03
ADT during RT			
No	1.0		
Yes	3.3	1.2–8.6	0.02
Final pathology Grade Group Gleason score			Overall *p* = 0.02
1	1.0		
2	0.28	0.05–1.45	0.13
3	1.11	0.25–4.92	0.89
4	0.38	0.05–2.69	0.33
5	1.33	0.27–6.65	0.73
pT stage			Overall *p* = 0.03
T1–T2a	1.0		
T2b–T2c	2.21	0.267–18.42	0.46
T3–T4	5.23	0.71–38.83	0.11
SVI			
No	1.0		
Yes	1.88	0.91–3.86	0.09
Positive surgical margins			
No	1.0		
Yes	2.04	0.99–4.2	0.052
ECE			
No	1.0		
Yes	1.99	0.99–4.14	0.06

*yrs* years, *cont* continuous, *PSA* prostate-specific antigen, *ADT* androgen deprivation therapy, *RT* radiotherapy, *SVI* seminal vesicle invasion, *ECE* extracapsular extension

However, pT stage and Grade Group categories, despite having statistically significant overall p-value, lost their significance when further subdivided within their subcategories (low sample size). Of note, the age, pre-radiotherapy PSA level, seminal vesicle invasion, extracapsular extension, and surgical margins status were not significantly associated with biochemical failure. We built multivariate prognostic model for PSA failure-free survival based on sufficient number of events (N = 30). Variables that remained significant on multivariate analysis were radiotherapy schedule (HFR vs CFR, HR 3.04, 95% CI 1.37–6.74, p = 0.006) and use of concomitant ADT (yes vs no, HR 4.41, 95% CI 1.6–12.12, p = 0.004).

Five-year metastasis-free survival was 76.9%. Among 16 patients who developed metastasis, nine patients (13%) developed metastatic castration-resistant disease and died of advanced prostate cancer.

Univariate analysis for metastasis-free survival is presented in Table 3. Variables found to be significantly associated with metastasis-free survival were the first postprostatectomy PSA (continuous variable), seminal vesicle involvement, positive surgical margins, and presence of extracapsular extension. Although Grade Group of the final pathology and pT stage had significant overall p-value, significance diminished after subdividing within subcategories. Patients with increasing level of the first postprostatectomy PSA, or with seminal vesicle involvement, or with positive surgical margins or with extracapsular extension found on prostatectomy specimen were at the higher risk of developing metastatic relapse. Since we observed 16 metastatic events, we decided to perform multivariable analysis and included all variables significant on univariate analysis as all p-values were very low. Variables that were found to be significantly associated with metastatic-free survival were the first postoperative PSA (HR 1.05, 95% CI 1.0–1.08, p = 0.0178) and presence of extracapsular extension (HR 4.99, 95% CI 1.23–20.25, p = 0.024), respectively.

**Table 3:** Univariate analysis for metastasis-free survival after salvage radiotherapy

Variable	Hazard ratio (HR)	95% CI	*p*-value
Age, yrs Cont	0.96	0.89–1.03	0.24
First post- prostatectomy PSA Cont	1.07	1.03–1.12	0.002
PreRT PSA Cont	1.07	0.98–1.16	0.053
Fractionation schedule			
66 Gy/33x	1.0		
52.5 Gy/20x	1.28	0.47–3.47	0.62
ADT during RT			
No	1.0		
Yes	7.83	1.0–59.3	0.05
Final pathology Grade Group Gleason score			overall *p* = 0.001
1	1.0		
2	0.0	0.01–14.9	0.945
3	0.32	0.06–1.56	0.16
4	0.14	0.01–1.52	0.11
5	1.06	0.2–5.57	0.95
pT stage			overall p = 0.002
T1–T2a	1.0		
T2b–T2c	55.08	0.002–64.8	0.96
T3–T4	49.9	0.004–56.1	0.95
SVI			
No	1.0		
Yes	3.48	1.26–9.60	0.02
Positive surgical margins			
No	1.0		
Yes	2.86	1.03–7.97	0.04
ECE			
No	1.0		
Yes	7.02	1.96–25.07	0.003

*yrs* years, *cont* continuous, *PSA* prostate-specific antigen, *ADT* androgen deprivation therapy, *RT* radiotherapy, *SVI* seminal vesicle invasion, *ECE* extracapsular extension

To better characterize prognostic value of the first postprostatectomy PSA level, we performed receiver operating characteristic (ROC) curve analysis to identify optimal cut-off point of persistently elevated PSA above which there is higher likelihood of metastatic relapse after salvage radiotherapy. Identified best cutoff point was PSA = 0.47 ng/mL, with Area under curve (AUC) of 0.749, sensitivity of 75%, and specificity of 69% (Fig. 3). This means that patients with the first postprostatectomy PSA > 0.47 ng/mL are at higher risk of developing metastatic disease after salvage radiotherapy.

### Treatment side-effects

After median follow-up of 67 months, in total four grade 3 side-effects were observed. In detail, one patient developed grade 3 severe symptomatic proctitis during the course of salvage radiotherapy which was interrupted. Three patients developed late grade 3 urinary side-effects: urinary obstruction required surgical urethrotomy, grade 3 hematuria requiring endoscopic coagulation and grade 3 urinary incontinence requiring artificial sphincter implantation. All patients received CFR. Interestingly, in hypofractionated arm no grade 2 or higher side-effects were noted. In all cohort, no grade 4 or 5 events were observed.

## Discussion

Presence of persistent PSA after radical prostatectomy rises dilemma whether the origin of PSA is local disease remnant after surgery, or the patient presumably has distant metastasis. In latter scenario, local radiotherapy would be omitted, and chance for cure would be lost, if the disease is present locally. Furthermore, such patients are underrepresented in relevant clinical trials as were mostly considered ineligible or outcomes for patients with persistent PSA were not reported (i.e. RADICALS, RAVES, GETUG-AFU17 trial). In postprostatectomy radiotherapy practice, these three landmark trials will define the care of patients after prostatectomy; so far they swung pendulum towards early radiotherapy salvage, however it is important to know that patients with multiple high-risk features were not considered eligible for those trials [21, 30, 31].

Experts estimate the incidence of persistent PSA after prostatectomy to be in the range 5–20%. In recent report by Preisser et al., from total of 11,604 patients who underwent prostatectomy in large tertiary center, 1025 (8.8%) had persistent PSA defined as PSA ≥ 0.1 ng/ml. Compared to patients who achieved undetectable PSA after surgery, patients with persistent PSA face increased risk of metastasis and dying from prostate cancer [9]. After propensity score matching, salvage radiotherapy improved both overall and cancer-specific survival in patients with persistent PSA. This paper highlights both the unfavorable nature of such patients and the need to administer salvage radiotherapy to improve outcomes. Moreover, in another study that analyzed outcomes of patients with detectable PSA post-surgery (defined as PSA > 0.1 ng/mL), biochemical progression was noted in 74% patients while 5% of patients developed metastases. Both PSA greater than 1 ng/mL and PSA velocity > 0.2 ng/ml per year were associated with higher likelihood of salvage treatment failure [32]. Furthermore, heterogeneity in salvage radiotherapy treatment outcome of patients with persistent PSA was confirmed in another study where salvage prostate bed radiotherapy initiation was associated with improved metastasis-free survival in patients with Gleason score ≤ 7 [33].

In our study, reported actual 5-year biochemical control in all cohort was 57% which is considerably favorable if we take into account high-risk population of treated patients with multiple adverse prognostic features. Fourteen (20%) of included patients in our study had the first postoperative PSA > 2 ng/mL (range 2.4–106.9 ng/mL). Among them, 11 patients experienced biochemical failure (median time to failure 41 months), and 7 patients developed metastasis (median time to metastasis 56 months). At the moment of database lockdown (January 2021) 3 patients are free of failure and had completed ADT.

Such patients with high PSA (i.e. > 2 ng/mL) were excluded from analysis of Gandaglia et al. as they were assessed to likely harbor metastatic disease [10]. However, we treated those patients despite high level of PSA as their staging investigations for metastasis were negative and they often had extensive local disease on MRI. To account for presumable occult metastatic disease in those patients we widely used ADT (68% of all treated patients received ADT).

Actual 5-year rate of distant metastasis in our cohort is 23% which is relatively high. In crude numbers, 16 patients developed distant metastasis (median time to metastasis was 40 months). Thirteen of them received ADT and their median time to metastasis was 42 months.

It is difficult to compare these results to available data in the literature given the lack of other studies addressing such unique population. To illustrate, Tendulkar et al. in their multi-institutional updated Stephenson nomogram predicting outcome of salvage radiotherapy did not include patients with persistent PSA. Overall, they reported 19% of 10-year cumulative incidence of metastasis for whole cohort. However, patients with PSA of more than 2.0 ng/mL had 37% 10-year metastatic rate compared to 9% for patients with PSA ≤ 0.2 ng/mL [23]. For all cohort, 5-year rate of PSA control was 57%. However, for patients with PSA of 0.51–1.0 ng/mL this rate was 54%. Interestingly, one third of patients with PSA of more than 2.0 ng/mL still had benefit from salvage radiotherapy. As described previously, patients with rising PSA after salvage radiotherapy were screened for metastases using bone scan, computerized tomography or choline PET/CT. Limitations of tools are well known. It is possible that using novel metabolic imaging we might have discovered more metastasis therefore we might underreported true metastatic relapse data for our cohort [34].

Important finding from our study is the fact that first postprostatectomy PSA level rather than pre-salvage radiotherapy PSA level had strong prognostic impact for success of salvage radiotherapy. Moreover, using ROC analysis, we identified cutoff level of PSA = 0.47 ng/mL which discriminates outcomes of distant control after salvage radiotherapy. More precisely, patients with first postoperative PSA ≥ 0.47 ng/mL have significantly higher likelihood of metastatic relapse following salvage radiotherapy compared to the patients with first postoperative PSA < 0.47 ng/mL. It can be hypothesized that postprostatectomy PSA provide some hints on the biology of underlying disease which are worth further exploring as has clear correlation with both PSA and metastatic failure.

Hypofractionation in postoperative setting after radical prostatectomy is appealing approach in terms of radiotherapy resource sparring, patient convenience and potentially improved (or at least non-inferior) efficacy. Several phase I and II trials are ongoing with reported only early toxicity and quality of life data. Example is phase I/II trial by Toronto group which included 30 patients and treated them with 51 Gy in 17 fractions (3 Gy daily fractions) to prostate bed. After median follow-up of 24 months authors reported 6% grade 2 or 3 toxicity, while 17% of patients experienced PSA failure. Authors concluded this hypofractionated regimen is well tolerated with encouraging PSA control, however their data are limited by short follow up [35]. Three additional innovative studies investigated hypofractionation in the postoperative setting. In study of Cuccia et al., 75 patients received prostate bed radiotherapy to a total dose of 63.8 Gy in daily fractions of 2.2 Gy (EQD2 67.4 Gy). In addition, 63% of patients received whole pelvis radiotherapy with median dose 49.3 Gy. After median follow-up of 30 months, authors reported 3-year biochemical control of 73% and 5.3% incidence of grade ≥ 2 late genitourinary toxicity [36].

In study of Fersino et al. 125 patients received moderate hypofractionation to prostate bed and pelvis using VMAT technique with median of 66 Gy to prostate bed and 52.5 Gy to the pelvis, delivered in 28 or 30 fractions. Only 1 event of grade 3 acute genitourinary toxicity was found with 77% 3-year biochemical control for salvage patients [37].

Finally, in study of Rigo et al., authors described salvage hypofractionated radiotherapy after primary HIFU failure in 24 patients. They used either 71.4 Gy/28 fractions or 32.5 Gy/5 fractions. After median follow-up of 28 months, they found local control achieved in 23/24 patients without serious toxicity [38].

Despite longer follow-up is needed to assess real value and role of moderate hypofractionation in postprostatectomy setting, these studies are foreshadowing new era in salvage radiotherapy brought by modern radiotherapy techniques which integrate physical and biological precision.

Although our study was not formally powered to compare CFR (66 Gy in 33 fractions) and HFR (52.5 Gy in 20 fractions), as the primary goal of the study was to report outcomes after salvage radiotherapy in high risk cohort with persistent PSA, fractionation schedule on univariate analysis did emerge as significantly associated with PSA failure-free survival. However, retrospective comparison of these two radiotherapy regimens further complicate more prevalent use of ADT with CFR, longer duration of ADT in CFR and baseline differences in Grade Group distribution between CFR and HFR patients. Therefore, our results on biochemical control associated with these two regimens need to be taken with grain of the salt. However, we need to acknowledge these two radiotherapy regimens differ significantly in terms of 2-Gy equivalent dose (EQD2). The respective EQD2 dose (alpha/beta = 3) for CFR and HFR group is 66 Gy, and 59 Gy, respectively, and are located on the steep part of dose–response curve. Lower biological dose delivered in HFR group hypothetically might be the reason for inferior biochemical control compared to CFR group. Conversely, higher biological dose in CFR group might well be the reason for higher incidence of grade 3 events in this patient group, which were not observed in HFR group.

Some groups advocate for even higher doses (i.e. > 66 Gy) in post-prostatectomy setting, specially in the context of modern radiotherapy techniques. Italian authors compared higher (≥ 70.2 Gy) and lower doses (< 70.2 Gy) of adjuvant postprostatectomy radiotherapy and found higher doses to be associated with improved biochemical control, however, mainly in patients with undetectable postoperative PSA [39]. In that regard, SAKK 09/10 trial prospectively compared salvage radiotherapy with 70 Gy in 35 daily fractions of 2 Gy and 64 Gy in 32 daily fractions of 2 Gy to prostate bed. After median follow-up of 6.2 years, there was no difference in freedom from biochemical progression between two arms, but higher dose arm had more late gastrointestinal toxicity [40]. It looks like that optimal radiotherapy dose in postprostatectomy setting is still matter of debate.

Anyhow, to properly test these two regimens in terms of efficacy and toxicity would require randomized trial with more than 1000 patients which is not likely to happen. Our findings add real-world experience on hypofractionation of salvage radiotherapy in specific patient population, i.e. those with persistent PSA after prostatectomy, a clinical context rarely reported in literature. Whilst CFR remains the standard option, in situations where due to other pressing factors a shorter course of radiotherapy is required, then HFR could potentially be considered, particularly in circumstances of global pressure on radiotherapy resources in COVID19 pandemic which will have lasting impact on clinical decision making in radiotherapy [41].

Limitation of the study include retrospective design, low sample size, imbalance of treatment factors and absence of prospectively assessed toxicity and patient-reported outcomes. Further studies, especially prospective ones, are welcomed to corroborate data on postprostatectomy hypofractionation and the role of persistent PSA in this setting.

## Conclusions

We showed that first post-prostatectomy PSA level, rather than pre-radiotherapy PSA level, is important prognostic factor for the success of salvage radiotherapy. Patients with first post-prostatectomy PSA > 0.47 ng/mL are at higher likelihood of metastatic relapse following salvage radiotherapy. Limitation of our study (inherent imbalances between treatment groups) preclude reliable efficacy comparison of two common salvage radiotherapy fractionation schedules.

## Data Availability

All data generated or analyzed during this study are included in this published article [and its supplementary information files].
